# Aberrant Activation of Immune and Non-Immune Cells Contributes to Joint Inflammation and Bone Degradation in Rheumatoid Arthritis

**DOI:** 10.3390/ijms242115883

**Published:** 2023-11-01

**Authors:** Kutty Selva Nandakumar, Qinghua Fang, Isabella Wingbro Ågren, Zoe Fuwen Bejmo

**Affiliations:** 1 Department of Medical Biochemistry and Biophysics, Karolinska Institute, 17177 Stockholm, Sweden; 2 Department of Environmental and Biosciences, Halmstad University, 30118 Halmstad, Sweden; isawin21@student.hh.se (I.W.Å.); zoebej21@student.hh.se (Z.F.B.); 3 Department of Urology, University of Pittsburgh School of Medicine, Pittsburgh, PA 15219, USA; hjfqh@163.com

**Keywords:** rheumatoid arthritis, inflammation, T cells, B cells, macrophages, fibroblasts, osteoclasts, autoantibodies

## Abstract

Abnormal activation of multiple immune and non-immune cells and proinflammatory factors mediate the development of joint inflammation in genetically susceptible individuals. Although specific environmental factors like smoking and infections are associated with disease pathogenesis, until now, we did not know the autoantigens and arthritogenic factors that trigger the initiation of the clinical disease. Autoantibodies recognizing specific post-translationally modified and unmodified antigens are generated and in circulation before the onset of the joint disease, and could serve as diagnostic and prognostic markers. The characteristic features of autoantibodies change regarding sub-class, affinity, glycosylation pattern, and epitope spreading before the disease onset. Some of these antibodies were proven to be pathogenic using animal and cell-culture models. However, not all of them can induce disease in animals. This review discusses the aberrant activation of major immune and non-immune cells contributing to joint inflammation. Recent studies explored the protective effects of extracellular vesicles from mesenchymal stem cells and bacteria on joints by targeting specific cells and pathways. Current therapeutics in clinics target cells and inflammatory pathways to attenuate joint inflammation and protect the cartilage and bones from degradation, but none cure the disease. Hence, more basic research is needed to investigate the triggers and mechanisms involved in initiating the disease and relapses to prevent chronic inflammation from damaging joint architecture.

## 1. Introduction

**Figure 1:**
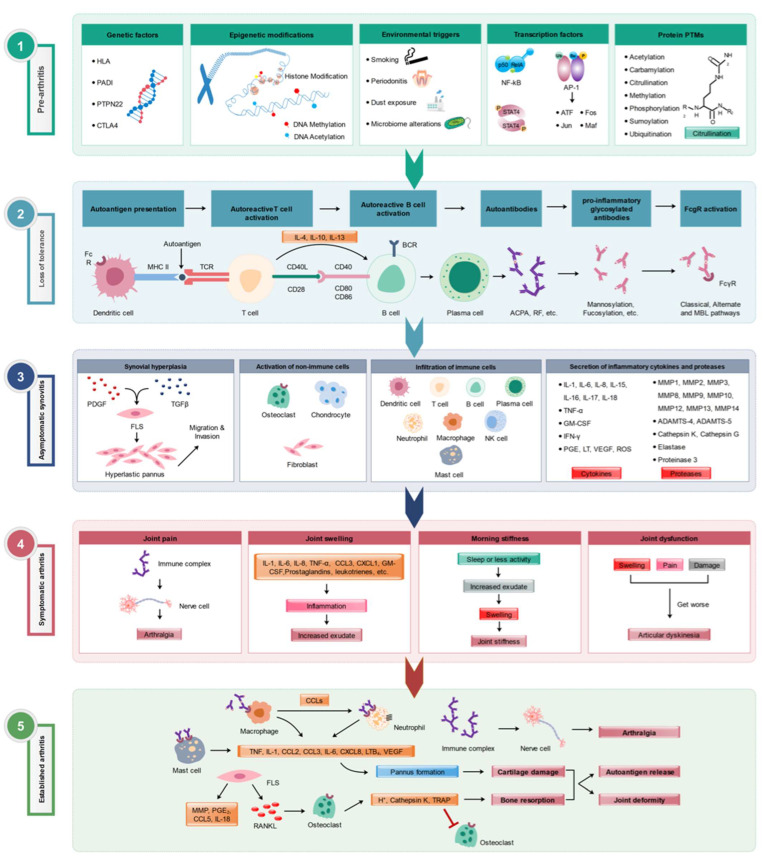
Various stages of RA development. Multiple factors involved in different (1) pre-arthritis, (2) loss of tolerance to self-antigens, (3) asymptomatic synovitis, (4) symptomatic clinical arthritis, and (5) established arthritis] phases of RA pathogenesis are depicted. Modified from [3].

Rheumatoid arthritis (RA) affects 0.5–1% of the population worldwide, in a female/male ratio of 3:1, and is most common among those aged 40–70. RA is primarily associated with inflammation within synovial joints. All peripheral joints can be affected in RA, but the most affected are those of the hands, feet, and knees [1]. Although RA’s etiology is still unknown, several factors contributing to RA have been identified. Among them are the susceptibility genes, disease-causing immune cells, and cytokine and signal transduction networks that promote inflammation (Figure 1). Various therapeutic strategies have been developed to target these factors, including TNF-α neutralizing agents, anti-IL-6, and B-cell-depleting antibodies [2]. Although none of these therapeutic strategies can cure the disease, some have proven more effective than others in ameliorating joint inflammation.

## 2. Pathogenesis and Diagnosis of Rheumatoid Arthritis

RA is a systemic, chronic, autoimmune disease categorized by synovial inflammation due to the infiltration of T cells, B cells, neutrophils, and macrophages, destroying articular joints and bone architecture. However, RA is not a homogenous disease but instead a syndrome of several sub-phenotypes. RA occurs when the body’s immune system attacks its proteins, so-called self-antigens. The pathogenesis behind RA is a combination of epigenetic, environmental, and genetic factors. Environmental factors contributing to immune system activation and inflammation in RA include smoking, microorganisms, and pollution. When these factors encounter mucous membranes, they can cause local inflammation and epigenetic modifications, including DNA methylation and acetylation [3]. Post-translational modifications (PTMs) of proteins could also occur due to environmental factors, which change a protein’s function and structure. For example, citrullination of proteins changes the protein’s structure, function, and interactions with the immune cells. Arthritis in the joint involves a multicellular inflammatory process involving infiltration of lymphocytes and granulocytes into the articular cartilage, proliferation of synovial fibroblasts and macrophages, and neovascularization of the synovial lining surrounding the joints. This proliferative process induces swelling, erythema, and pain in multiple joints, leading to destruction and loss of bone density and architecture.

Our body initiates the joint-specific attack by producing autoantibodies [4,5] because of aberrant activation of the B cells recognizing either a self- or cross-reactive antigen. The antibodies, after binding to the joint-specific antigens like collagen type II, present abundantly in the articular cartilage, and other cartilage matrix proteins (cartilage oligomeric matrix protein, collagen type XI) deposited on the cartilage surface could activate the complement system and attract phagocytes to the inflammation site. Active immunization of mice with these cartilage matrix proteins or passive transfer of antibodies specific to them induced arthritis. In addition, serum from K/BxN mice containing anti-glucose-6 phosphatase isomerase (GPI) also induced arthritis in mice due to the binding of these antibodies to GPI deposited on the cartilage surface. Because of the increased immune attack on the joints by the effector cells, there can be a great deal of damage to the articular joints by further activating the complement system through three different pathways (classical, alternative, and lectin pathways) involving several proteases [6]. Mainly, the alternative pathway is involved in activating the terminal pathway. Thus, therapeutic efforts in RA might be improved if the treatment includes its inhibition [7] and the Fc-gamma (FcγRs)-bearing immune effector cells [8,9], like macrophages, dendritic cells, neutrophils, and others. This makes an early diagnosis more crucial for providing optimal therapy [4]. Relative contributions of complement and FcR-mediated inflammatory pathways to the antigen–antibody complex-dependent inflammatory responses could vary depending on many factors. These include antibody isotype and titer and the site of immune complex deposition. Concerning the Ig isotype, FcγR mechanisms could predominate with immune complexes comprised of non-complement-fixing antibodies or deposit in the locations with abundant resident FcγR-bearing inflammatory cells. Conversely, complement-driven inflammation may dominate when FcγR poorly binds immune complexes containing Ig-constant regions or when leukocytes must be attracted to an inflammatory site.

Many disease-specific autoantibodies are present in different autoimmune diseases. In arthritis, autoantibodies target antigens with a variety of post-translational modifications like citrullination, carbamylation (a non-enzymatic, post-translational modification binding to isocyanic acid), and acetylation [10]. Citrullination might be a prerequisite for triggering the generation of ACPAs in susceptible RA patients [11,12,13]. Citrullinated autoantigens, abundant in RA synovial tissue, play a central role in the disease process. These are altered forms of proteins that arise due to post-translational modification involving the conversion of arginine to citrulline [14]. The citrullinated autoantigens can be recognized by antigen-presenting cells (APCs) like macrophages, neutrophils, and dendritic cells and present the antigen on their cell surface in conjunction with the HLA and travel to the draining lymph nodes where the APCs activate the T cells. Activation of self-reactive T cells triggers a cascade of immune events that contribute to the chronic inflammation and tissue damage observed in RA [15], which includes signaling molecules and cytokines like IFN-γ, IL-4, IL-10, and IL-13, stimulating B cells to differentiate into plasma cells to secrete autoantibodies.

ACPA targets are present in the synovium and synovial fluid [16], cartilage [17], lungs [18], and inflammatory cells like neutrophils and macrophages [19,20]. The development of autoantibody characteristics like the use of different isotypes, recognition of specific epitope(s), antibody avidity [21], and affinity, as well as the glycosylation status, reveal their relationship to RA risk factors and clinical phenotypes [10]. The HLA-DRB1 shared epitope, PTPN22, and smoking are associated with specific ACPA reactivities rather than anti-CCP levels [22]. A combination of smoking and HLA-SE alleles is significantly related to the development of some of the ACPA specificities closer to the onset of clinical symptoms, and these associations remained significant at diagnosis [23].

Interestingly, ACPAs can be detected years before the disease onset in a subgroup of patients, and at the onset of clinical symptoms, a broad isotype spectrum is observed [24,25]. Epitope spreading with increased recognition of citrullinated antigens occurs before the onset of RA [26]. An association of valine and leucine at HLA-DRB1 position 11 with radiographic progression in RA was reported to be independent of the shared epitope alleles but not ACPAs [27].

Similarly, RANKL (receptor activator of nuclear factor kappa-B ligand) concentrations are increased several years before the onset of arthritis symptoms, particularly in ACPA/RF/anti-CarP-positive individuals, all detectable earlier than RANKL. Positivity for RANKL and anti-CarP antibodies yielded the highest Larsen score in disease onset [28]. Combinations of IgM and IgA isotypes with ACPA specificities, including α-enolase (CEP-1), fibrinogen (Fib)β36-52, Fibα580-600, filaggrin (CCP-1), and anti-CCP2 antibodies, are associated with a shorter time to onset of clinical symptoms [29]. ACPAs are modified both in the constant and variable domains by the presence of N-linked glycans.

Recently, the presence of citrullinated autoantigens in platelets and the platelet-derived microparticles (PDPs) are identified as the potential targets of ACPAs in RA. Citrullinated forms of thrombospondin-1, β-actin, and platelet factor-4 (also known as CXCL4) are highly immunogenic and recognized by ACPAs [30]. ACPAs are associated with a more destructive disease phenotype, although their functional role might be pathogenic and protective depending on their specificity [31]. Previously, anti-citrullinated vimentin antibodies were shown to induce osteoclastogenesis and bone loss [32]. ACPAs can be of great help in identifying the disease activity in the early development of arthritis to treat the patients at an optimal period to prevent a more relapsing and chronic disease pattern, as observed in collagen-induced arthritis, a classical animal model of RA, with the development of epitope-specific, anti-collagen type II (CII) antibodies [33]. We have shown that autoantibodies specific to the CII triple-helical epitope bind and destabilize the cartilage independent of inflammation by an impairment of matrix synthesis on chondrocyte cultures with adverse effects on preformed cartilage and a significant proteoglycan depletion even in the absence of complement factor C5 [34]. Autoantibodies form immune complexes in the joints that attract immune effector cells [35], which initiate cytokine release and prolongs B cell survival pathways. Notably, the increased serum concentration of RF indicates a more aggressive articular disease and RF levels can help diagnose different subtypes of RA [4].

Glycosylation is a post-translational modification that affects most of the antibody’s mediated functions. FcγRs and complement binding to differentially glycosylated antibodies have altered functions like cellular cytotoxicity, phagocytosis, and cytokine secretion. Current studies illustrate that appropriate modification of Fc glycosylation of antibodies could attenuate the effector phase of arthritis and potentially be a future treatment option. In this context, we have identified the importance of IgG Fc-glycosylation in arthritis. Streptococcal Endo-β- *N*-Acetylglucosaminidase (EndoS) secreted by *Streptococcus pyogenes,* which hydrolyses the β-1,4-di-*N*-acetylchitobiose core of the N-linked complex type glycan on the asparagine 297 of the γ-chains of IgG, induced suppression of local immune-complex-mediated arthritis. EndoS disturbed more extensive immune complex lattice formation on the surface of joints, as visualized using anti-C3c staining. Neither complement binding nor antigen–antibody binding per se was affected by the removal of IgG N-glycans by EndoS [36], and this enzyme treatment abolished the arthritogenicity of autoantibodies [37].

The IgG Fc part acts as a bridge between the innate and adaptive immune responses. It is regulated by N-glycosylation present in the variable structures, including agalactosylated and galactosylated forms, which are further modified by fucosylation and bisecting N-acetylglucosamine moieties. The bisecting proinflammatory N-acetylglucosamine moiety G0F was shown to be differentially regulated by estrogen via IgG glycosylation [38]. Similarly, phytoestrogens were found to protect the joints in experimental arthritis by increasing IgG glycosylation and reducing osteoclast activation [39]. The IL-23/T_H_17 axis could be a decisive factor in controlling the intrinsic inflammatory activities of autoantibodies, which trigger the clinical onset of arthritis [40].

## 3. Activation of Immune Cells in RA

Several immune cells (T cells, B cells, monocytes/macrophages, dendritic cells, neutrophils, etc.) in the synovium and circulation contribute to disease pathogenesis in RA. Infiltration of T and B cells in the inflamed joints and the observed association of specific human leukocyte antigen (HLA) alleles suggest the importance of adaptive immunity in RA development [41]. DR genes, including DR4 and DR1 (for example, DRB*0401, DRB*0404, and DRB*0101), are associated with RA. The susceptibility epitope is glutamine–leucine–arginine–alanine–alanine or QRRAA, the shared epitope. T cells, by secreting proinflammatory cytokines, activate the non-immune cells like synovial fibroblasts (type A synoviocytes) and macrophages (type B synoviocytes) into a more aggressive phenotype, causing “pannus” formation and promoting further secretion of cytokines and chemokines leading to joint inflammation. Whereas B cells, apart from activating T cells and secreting proinflammatory cytokines, differentiate into plasma cells that secrete different types of antibody-recognizing self-antigens such as rheumatoid factors, anticitrullinated protein/peptide antibodies, anti-glucose-6-phosphate isomerase antibodies, anti-CII-specific antibodies, and so on. It is well established that some of these autoantibodies are used as diagnostic markers, and their pathogenic nature was also proved using animal models [42,43]. As described earlier, RA is a disease with a complex pathogenesis dependent on several environmental, epigenetic, and genetic factors. Although several tolerance mechanisms at T and B cell levels can limit autoimmune responses, the breakdown of such mechanisms results in autoimmune diseases. However, using anti-inflammatory extracellular vesicles from mesenchymal stem cells at the T and B cell levels [44] provides a more promising approach to control inflammation in RA.

### 3.1. B Cells

The contribution of B cells to RA development is both antibody-dependent and independent. Rheumatoid factors (RF), comprising IgM, IgG, and IgA class antibodies recognizing the Fc part of the IgG antibodies (though not specific for RA), anti-citrullinated protein/peptide antibodies (ACPAs, highly specific for RA) [45,46], and anti-CarP antibodies binding to carbamylated antigens [47] and other autoantibodies, are present in the sera and synovial fluids of RA patients. Similarly, antibodies to other post-translationally modified peptides/proteins like malondialdehyde (MDA)–acetaldehyde (MAA)–lysine, and acetylated proteins are primarily present in ACPA-positive RA patients. Some of these antibodies, like RF [48], anti-CarP [47,49], and ACPAs [24,25], are produced and in circulation before the onset of clinical disease [50]. High serum levels of RF can predict a more severe form of the joint disease [51]. Notably, the specificity of ACPA positivity has high sensitivity (60–78%) and specificity (86–99%) values [52,53]. ACPAs are strongly linked to the HLA class II locus, suggesting their involvement in RA development [54]. During citrullination, peptidyl arginine is converted to citrulline by the Ca^2+^-dependent enzyme peptidyl arginine deiminase (PAD). Various forms of PADs were reported that differ in their location and functional activities. PAD2 and PAD4 play a critical role in RA development by generating citrullinated antigens and contributing to neutrophil extracellular trap (NET) formation by citrullinating nuclear proteins [55,56].

Defects in the B cell tolerance mechanisms allow increased levels of autoreactive B cells, possibly contributing to disease development in the RA [57]. Many tolerance mechanisms operate at the B cell level to prevent autoimmunity [58]. At the central level, self-reactive B cells are eliminated via clonal deletion [59,60], receptor editing [61,62], and clonal anergy [63] mechanisms. As reported earlier, B cells that escape tolerance induction can undergo somatic mutation and affinity maturation in the periphery, causing pathogenicity [64] by generating autoantibodies. Using RF transgenic mice, Hannum et al. [65] have shown that autoreactive B cells induced normal immune responses and were not anergic.

Similarly, using a germline-encoded CII IgH transgenic mouse model, autoreactive B cells to CII were found to be neither negatively selected nor tolerized but spontaneously produced autoantibodies without any disease development [66]. However, introducing a gene mutation causing reactive oxygen species deficiency led to disease development with an increased germinal center (GC) formation, T cell response, and epitope spreading [67]. Later, GC formation and anti-CII antibody production were identified as the primary pathogenic functions of B cells in the collage-induced arthritis model [68]. These studies suggest the presence of similar pathogenic B cells in humans, which, along with autoreactive T cells, when activated under specific conditions, could contribute to disease development. Kristyanto et al. have shown that sustained activation of expanding memory self-reactive B cells promoted inflammation in RA [69].

Moreover, autoreactive B cells can also present antigens by expressing co-stimulatory molecules to stimulate T cell maturation and differentiation [70] and contribute to synthesizing not only proinflammatory [71] but also regulatory cytokines such as IL-1, IL-4, IL-6, IL-8, IL-7, G-CSF, GM-CSF, IL-10, IL-12, and TGF-β which can be involved in immune responses [72]. By regulating T cells, B cells can modulate the activity and differentiation of these cells. When B cells present autoantigens to T-helper cells, this interaction can activate self-reactive T cells, ultimately influencing the immune responses. Activation of self-reactive T cells produces signaling molecules, including cytokines like IL-4, IL-10, and IL-13, that stimulate B cells to differentiate into plasma cells to secrete autoantibodies causing damage to bone and cartilage. In this context, more autoreactive B cells were reported earlier in RA patients due to a compromise in early B cell tolerance mechanisms [57]. Of note, B1a cells secrete natural autoantibodies without external antigen stimulation. These act as scavengers and help to remove autoantigens and apoptotic cells. However, B1a cells were shown to be pathogenic in the CIA mouse model [73].

Interestingly, biased V-region gene usage and conserved junction arrangements in B cell receptors from RA were reported, suggesting broad ACPA specificities found in RA were from a restricted repertoire of citrulline-specific polyreactive B cell lineages [74]. ACPAs from an individual RA patient can recognize different citrullinated antigens [75,76]. Human filaggrin, α-enolase, fibrinogen, fibronectin, vimentin, collagen II, immunoglobulin-binding protein (BiP), and histone proteins are some of the citrullinated targets for autoantibodies from RA patients [12,52,76,77,78,79,80,81,82,83]. Significant epitope spreading of ACPAs before the onset of joint inflammation is well documented [26,84,85,86,87]. These autoantibodies form immune complexes, attracting immune effector cells and activating complement cascades [35]. In this context, ACPAs were known to activate the complement and Fcγ-receptor-bearing immune cells [88]. However, not all the ACPAs are pathogenic [89].

Depletion of B cells using anti-CD20 monoclonal antibodies (rituximab), possibly through complement-dependent cytotoxicity (CDC) and antibody-dependent, cell-mediated cytotoxicity (ADCC), proved to be effective in a subset of RA patients [90,91]. However, autoantibody levels were not significantly reduced [92], suggesting antibody-independent functions of B cells in RA-like antigen-presentation to T cells [93], secretion of proinflammatory cytokines, regulation of the functions of T cells [94], facilitation of lymphoid tissue organization during synovitis [95], and the activation of osteoclasts by secreting receptor activator of nuclear factor kappa-B ligand [96] also contribute to disease development. A comprehensive transcriptomic analysis of citrulline-specific B cells from RA patients showed a differential expression of the IL15 receptor alpha gene and other genes related to protein citrullination and cyclic AMP signaling [97]. In addition, these cells produced amphiregulin, which is involved in an increased migration and proliferation of synovial fibroblasts. Together with ACPA antibodies, they induced the differentiation of osteoclasts [97]. Approximately 5% of sub-lining synovial cells are B cells, which undergo clonal expansion in the joints during RA development, suggesting antigen-driven maturation processes. Autoantibody production occurs in the joints of many RA patients, which can contribute to joint pathology in situ by activating the complement system, producing the anaphylatoxins (C3a and C5a) and attracting FcγR-expressing immune cells like macrophages and neutrophils secreting various proteases, radicals, and proinflammatory factors. In addition, memory B cells from RA patients’ blood and synovial fluid tissues showed an increased RANKL production, which could contribute to enhanced osteoclast activation [96] and joint destruction.

### 3.2. T Cells

Several pieces of evidence point to the crucial contribution of T cells in RA pathogenesis. The most vital genetic link to RA is the HLA class II involved in antigen presentation to T cells [98,99,100]. Moreover, RA-associated allelic variants of several genes like protein tyrosine phosphatase non-receptor type 22 (PTPN22), cytotoxic T-lymphocyte-associated protein 4 (CTLA4), the zeta chain of T cell receptor-associated protein kinase 70 (ZAP70), and peptidyl arginine deiminase 4 (PADI4) encode molecules involved in the T cell activation pathways. CD4+ T cells are among the infiltrating inflammatory cells in the synovial tissue and promote activation of the resident synovial cells (fibroblasts and macrophages) by secreting proinflammatory cytokines. Strong evidence for T cell involvement in disease development has emerged from the analysis of RA synovial biopsies [101]. A study of synovial tissues from ACPA-positive patients showed an elevated level of infiltrating lymphocytes associated with ectopic GC formation [102].

**Figure 2:**
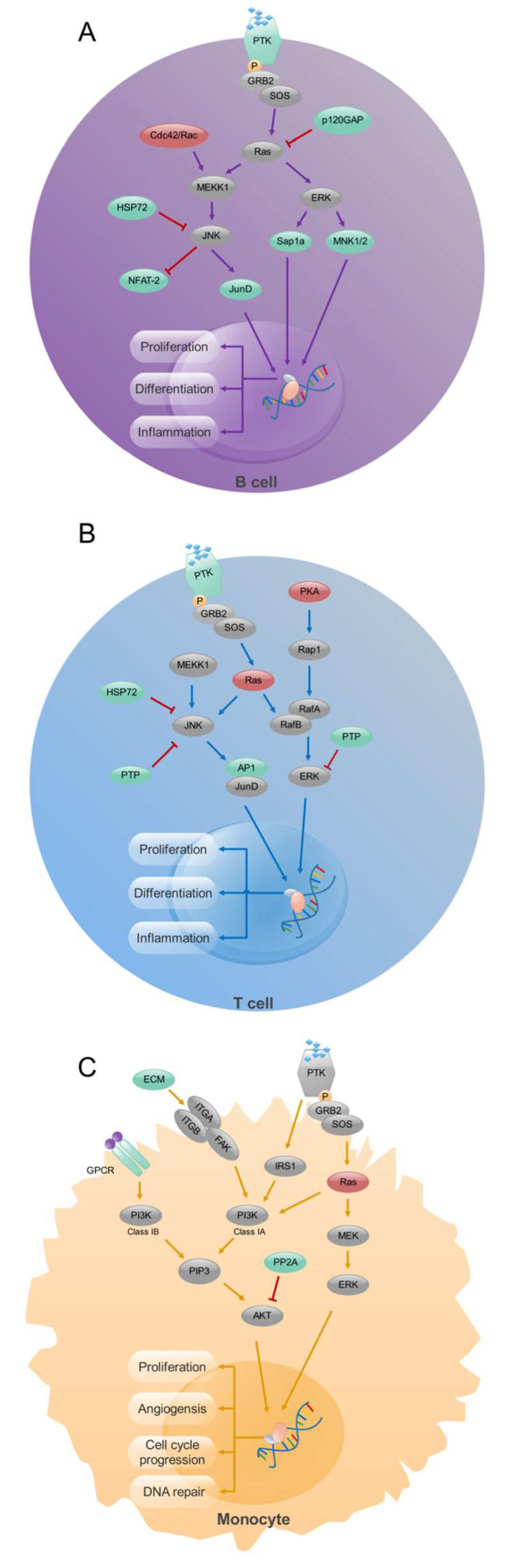
Signaling pathway analysis in CD19^+^ B- cells, CD4^+^ T- cells, and CD14^+^ monocytes from RA patients. Enrichment of MAPK signaling pathway in CD19^+^ B (**A**) and CD4^+^ T (**B**) cells and PI3K signaling pathway in CD14^+^ monocytes (**C**). Up-regulated (red), down-regulated (green), and unchanged (gray) genes are marked in the figure. Modified from [113].

Expression of specific transcription factors stimulated by various cytokines determines the differentiation of Th cells into different subsets (T-bet for Th1 [103]; GATA3 for Th2 [104]; RORγt for Th17 [105]; and BCL6 for T follicular helper cells [106,107,108]). Therefore, these transcription factors could very well be contributing to RA pathogenesis. FoxP3 is crucial for the differentiation and function of Treg cells, which control aberrant activation of T cells; depletion of Treg cells promotes systemic autoimmunity [109]. Notably, a balance between these T cell subsets is critical for the homeostasis of immune functions, and disturbance to this balance was found in RA [110]. A specific deficiency of CTLA-4 in Tregs results in severe autoimmune responses in mice [111]. B-cell-induced LAG3(+)FOXP3(−) regulatory T cells alleviated joint inflammation in collagen-induced arthritis [112]. Using a combined analysis of gene expression and methylation profiling microarray in immune cells of RA, in CD4^+^ T cells, we identified exceptionally methylated, differentially expressed genes (DEGs) enriched in the processes of formation of new blood vessels, transcription of genes, transport of molecules, and cell shape regulation. Further, our study suggests that the MAPK signaling pathway could differentiate the mechanisms affecting T and B cells in RA (Figure 2) [113]. Therefore, MAPK phosphatases could be novel targets for attenuating inflammation in RA [114].

The T cell cytokine IL-17 is highly produced by RA synovium [115], and increased levels of Th17 cells [116] and Tfh cells supporting high affinity and long-term antibody responses correlating to disease activity [117] were identified in the blood samples from RA patients. Increased PD-1^high^CXCR5-CD4+ T cells in the synovium of RA patients specifically promoted B cell responses and the generation of antibodies within inflamed nonlymphoid tissue in RA patients [118]. Other disease-relevant T-cell subsets (effector memory-Tfh and T helper 17) and disease-driving pathways like mTORC1, IL-2-STAT5, cell cycle, E2F, and interferon-related genes, several proinflammatory cytokines, and chemokines involved in RA pathogenesis were also reported earlier [119]. B cells and macrophages present disease-related antigens to T cells to secrete cytokines [120], activating chondrocytes and osteoclasts to produce matrix lysing enzymes, leading to cartilage degradation and bone resorption [121].

### 3.3. Monocytes/Macrophages

Macrophages play a crucial role in the chronic inflammation and progressive joint destruction in RA. The resident macrophage-like synovial cells in the joint intimal lining layer are of monocyte–macrophage lineage, originating from the bone marrow [122]. Synovial macrophages, primarily of the phenotype known as M1, are characterized by their elevated levels of proinflammatory proteins, such as prolyl hydroxylase 3 (PHD3), matrix metalloproteinase 12 (MMP12), C–C chemokine receptor type 2 (CCR2), and tumor necrosis factor-alpha (TNF-α) [3]. M1 macrophages also secrete a variety of proinflammatory cytokines (e.g., IL-6, IL-12, and IL-1β) as well as chemokines (e.g., CCL2 and CCL5) that contribute to joint tissue damage [123]. These proinflammatory M1 cells promote inflammation and neutrophils and recruit immune cells such as monocytes, neutrophils, and lymphocytes to the affected joints, activating fibroblasts and osteoclasts, thereby collectively triggering a cascade of damaging inflammatory reactions within the synovium.

Numerous macrophages are present in the inflamed synovial membrane and at the cartilage–pannus junction [124], possibly due to defects in apoptosis. NF-κB activation [125] and signaling through the PI-3K-AKT pathway [126] have been shown to inhibit apoptosis. FLIP (FADD-like IL-1β-converting enzyme-inhibitory protein) is highly expressed in RA synovial macrophages, which prevents Fas-mediated apoptosis [126]. In addition, the synovial environment is hypoxic, and low pO2 is a potent inhibitor of apoptosis. In RA joints, macrophages show over-expression of MHC class II and production of proinflammatory cytokines (IL-1β, TNF-α, GM-CSF, and IL-6), chemokines (IL-8, MIP-1α, and MCP-1), metalloproteinases, and neopterin [127], which could drive the disease progression. Furthermore, monocytes present antigens to T cells and regulate their differentiation and functions, contributing to their immune and inflammatory responses. In the synovium, monocytes differentiate into macrophages and osteoclasts, causing the production of proinflammatory factors and bone destruction [128]. Macrophage depletion from inflamed tissues seems to have therapeutic benefits in RA [129]. The macrophage-like synoviocytes present in the joints of RA patients produce several proinflammatory cytokines, including IL-1, IL-6, TNF-α, and other inflammatory factors contributing to cartilage and bone damage. Activation of macrophages by proinflammatory cytokines, antigen-antibody complexes, and TLR agonists [130] is a crucial step in the pathogenesis of RA. While identifying abnormally methylated DEGs and pathogenic mechanisms in monocytes via comprehensive bioinformatics analysis, we found enrichment of the PI3K pathway in the RA [113]; therefore, PI3K Inhibitors could also be helpful in the treatment of inflammation and autoimmunity [131].

During inflammation, proinflammatory M1 type macrophages and Th17 cells show metabolic changes by shifting toward increased uptake of glucose, breakdown of glucose to produce energy, and use of the pentose phosphate pathway generating NADPH and non-oxidative synthesis of pentoses, the five carbon-containing sugar molecules. In contrast, anti-inflammatory cells like M2-type macrophages, regulatory and memory T cells display a lower level of glycolysis but with an increased state of oxidative metabolism [132]. Two lineages of synovial macrophages in the mice were identified earlier: intrinsic macrophages expressing anti-inflammatory cytokines and extrinsic macrophages expressing proinflammatory cytokines [133]. A similar population of cells has also been identified in patients with RA. Therefore, changes in the immune metabolism suggest possible new therapeutic approaches targeting the proinflammatory metabolic pathways of macrophages.

### 3.4. Dendritic Cells and Neutrophils

In RA, dendritic cells (DCs) take on a multiplex role, contributing to the autoimmune process of the disease. During regular homeostasis, DCs maintain immune regulation, whereas, during RA, DCs can trigger the differentiation and activation of autoreactive T cells by presenting self-peptides on their cell surface and triggering innate immune functions. This can drive the inflammatory cascade, contributing to the chronic inflammation in RA. Metabolism has a decisive role in activating the conventional dendritic cells in the RA synovium that have an increased capacity to migrate and activate T cells, and the plasmacytoid dendritic cells that could be tolerogenic [134].

Similarly, neutrophils have been found to have increased inflammatory activity and oxidative stress in RA. Neutrophils release neutrophil extracellular traps (NETs), which in turn interact with fibroblasts-like synoviocytes (FLS), contributing to proinflammatory cytokine release, generation of autoantibodies, and antigen presentation [135]. Elevated concentrations of NETs have been identified in the serum, synovial tissue, rheumatoid nodules, and skin of ACPA-positive RA patients. The activated neutrophils ingest immune complexes, releasing a spectrum of proteases and oxidative radicals that induce cartilage matrix degradation in the joints [136].

### 3.5. Non-Immune Cells in RA

**Figure 3:**
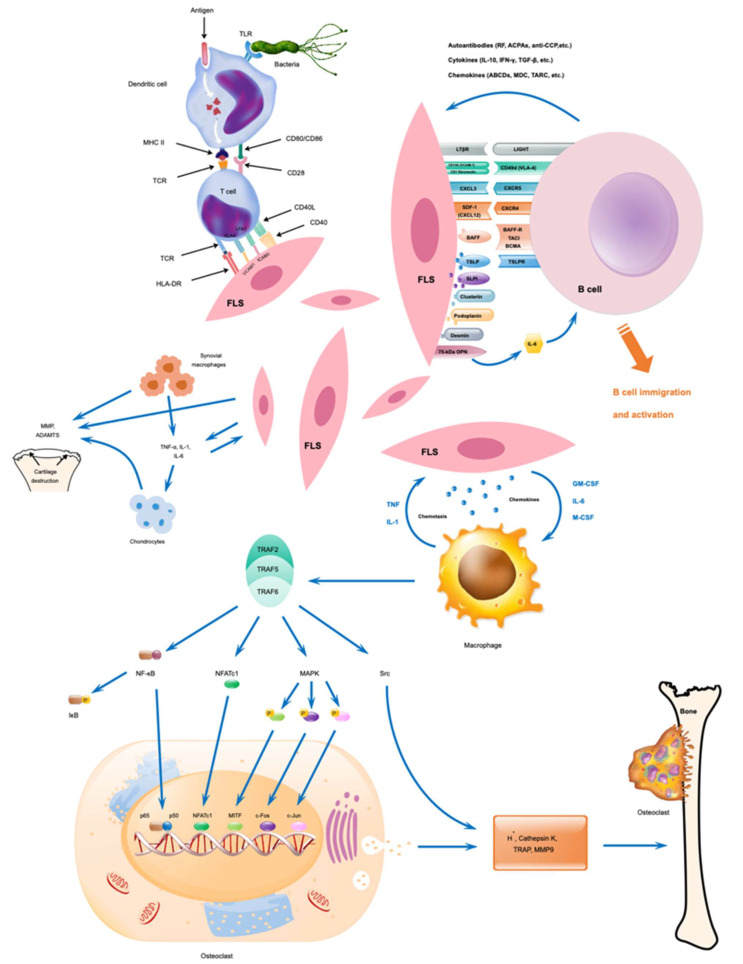
Possible interactions between immune and non-immune cells and their activation during RA development. Modified from [3].

Chondrocytes, fibroblasts, and osteoclasts are the primary non-immune cells that can contribute to the pathogenesis of RA (Figure 3). Chondrocytes, originating from bone marrow mesenchymal stem cells (MSC), produce collagen and extracellular matrix (ECM) components and are responsible for the maintenance of cartilaginous matrix and articular cartilage development. Chondrocyte functions are regulated by various cytokines and cellular signals [137]. However, under inflammatory conditions, activated chondrocytes participate in the degradation of cartilage and release of cytokines into the synovium, causing increases in the catabolic reactions and proinflammatory processes [3]. Autoantibodies affect chondrocytes in the cartilage by activating them, causing degradation, and releasing cytokines into the synovium. This leads to increased catabolic reactions and proinflammatory processes that worsen the state of RA via the breakdown of cartilage with a positive feedback loop. For example, autoantibodies binding to a specific epitope of CII were shown to impair matrix synthesis in chondrocytes [34] and another monoclonal antibody-induced abnormal chondrocyte morphology [138] demonstrating antibody-mediated effects on chondrocytes. However, extracellular vesicles from MSC can rescue the disease phenotypes of chondrocytes [139].

Fibroblasts-like synoviocytes (FLS), a type of specialized mesenchymal cells, contribute substantially to RA development by secreting several inflammatory cytokines like TNF-α and IL-17, matrix-lysing enzymes, and chemokines under inflammatory conditions, which confer an aggressive and invasive phenotype to them. FLS, after being stimulated by IL-17, transforms into a hyperactive form, aiding RA progression. FLSs become tumor-like in their phenotype due to epigenetic changes, and secrete cytokines and proteases that mediate the destruction of cartilage and further RA progression. These FLSs transform the synovium into a hyperproliferative, invading pannus-like structure, secreting inflammatory molecules, perpetuating inflammation, and destroying joint cartilage, bone, and ECM [140]. Glycolysis is activated in RA-FLSs because of the ongoing inflammation, and targeting the glycolytic pathway could plausibly reduce inflammation [141]. Earlier, we have shown that metformin, an AMP-activated protein kinase (AMPK) activator, blocked FLS expansion, dispersion, and stimulation [142]. In addition, metformin inhibited the expression of inflammatory cytokines while increasing the hyaluronan and proteoglycan link protein 1 (HAPLN1) expression [142]. Later, HAPLN1, which contributes to the stability of ECM and compression resistance of the joints [143], was found to promote the expansion and inflammatory phenotype of FLS [144].

T cell cytokines such as interleukin IL-2 and interferon IFN-γ are in relatively low concentrations. In contrast, macrophage and fibroblast products were abundant [145], including IL-1, IL-6, IL-15, IL-18, TNF-α, GM-CSF, various chemokines, and many others, which are produced by rheumatoid synovium. The effector cytokines, TNF-α, and IL-1β levels strongly correlate with disease symptoms and joint damage in RA [124]. Moreover, IL-1 increases the production of factors that stimulate cartilage matrix degradation and could inhibit the synthesis of CII and proteoglycans, significant proteins in the articular cartilage. Suppressive cytokines, such as transforming growth factor-β (TGF-β) and IL-1 receptor antagonist, as well as anti-inflammatory cytokine signaling mechanisms, such as the suppressor of cytokine signaling 3 (SOCS3), are expressed in RA synovium but at levels that are inadequate to block synovitis [146].

Osteoclasts, formed after fusion and differentiation of mononuclear precursor cells, could resorb bone matrix by producing various proteases [147]. Both TNF-α and IL-1β have been implicated in the dysregulation of bone and cartilage remodeling, characteristic of RA, by up-regulating the production of RANKL, which enhances osteoclastic bone resorption. In addition, TNF-receptor-associated factor (TRAF) activates the osteoclasts to degrade the bones. TNF-α stimulates the differentiation of osteoclast progenitors into mature osteoclasts, and IL-1 acts directly on osteoclasts to increase the bone-resorbing capacity of these cells. Osteoclast numbers are regulated by maintaining a balance between differentiation and death. Hence, bone loss could be prevented by reducing their formation and increasing the rate of cell death. Both programmed cell death [148] and microRNA [149,150,151] can regulate bone homeostasis. Several miRNAs were shown to be involved in the formation and maturation of osteoclasts, apoptosis, and their bone resorptive functions. So, inhibiting osteoclastogenesis and promoting osteoclast apoptosis might improve bone destruction. Recently, we reported the osteoprotective functions of a pathogenic bacterium, *Proteus-mirabilis*-derived outer membrane vesicles (OMVs). *P. mirabilis* OMVs inhibited the expression of miR96-5p, leading to an increase in ATP binding cassette subfamily A member 1 (Abca1) and mitochondria-dependent apoptosis in osteoclasts by enhancing the level of intracellular ROS and disintegration of mitochondrial membrane potential. Thus, modified OMVs with reduced toxicity and miRNAs could plausibly be used to treat osteolytic patients [152]. The role of intestinal microbiome, therapeutic modulation, and contribution of prebiotics and probiotics in RA therapy were discussed earlier [153,154,155].

RA patients are generally treated with disease-modifying, anti-rheumatic drugs (DMARDs), which function via immunosuppression and immunomodulation. Conventional DMARDs include the most-used methotrexate, hydroxychloroquine, leflunomide, sulfasalazine, azathioprine, and cyclosporine. At the same time, biological DMARDs like infliximab, adalimumab, etanercept, rituximab, abatacept, tocilizumab, tofacitinib, and others are used more specifically to target an immunological pathway. A combination therapy with a biological and a conventional DMARD has shown to be a more promising treatment than monotherapy to attenuate inflammation and reduce long-term joint damage. However, continuous use of DMARDs could cause several side effects, including an increased risk for infections, abdominal pain, liver problems, GI disturbances, etc. Nonsteroidal anti-inflammatory drugs (NSAIDs) are commonly used in the treatment of RA to manage pain, inflammation, and swelling, but they do not significantly affect the progression of the disease. Although the potential side effects of glucocorticoids for treatment in RA preclude its frequent use, because of their anti-inflammatory properties and the capacity to decrease radiologic progression in early RA, the European League Against Rheumatism and the American College of Rheumatology recommend glucocorticoids as an adjunct treatment to conventional synthetic DMARDs, at the lowest dose for the shortest time. Therefore, it is crucial to detect RA at an early stage for treatment to prevent the radiographic progression of the disease.

## 4. Conclusions

Genetic and environmental factors contribute to the development of RA. Smoking, infections, and pollutants could cause mucosal inflammation, inducing epigenetic modifications and post-translational modifications of proteins before the induction of clinical disease in genetically predisposed individuals. The breakdown of immune tolerance mechanisms leads to an aberrant activation of immune cells, production of cytokines, and autoantibodies, which in turn activates more immune and non-immune cells. Autoantibodies in the form of immune complexes activate the immune system further and are identified as one of the contributors to the development of pain, cartilage destruction, and bone damage. If the damage is minor and transient, disease resolution occurs, but in untreated patients or under constant stimulation, chronic inflammation persists, which damages the joints, causing disability.

The principal site of inflammation in arthritis is the synovial membrane, where the residential FLS and synovial macrophages, upon aberrant activation, secretes cytokines and enzymes destroying cartilage. Alterations in the metabolic pathways in these cells were observed. Therefore, targeting them could attenuate inflammation and damage to joints in RA. The issue is the specificity in targeting the metabolic pathways only in specific inflammatory cells. However, it is still possible to deliver drugs to individual cells by conjugating them to antibodies or antibody-coated nanoparticles. Therapies targeting synovial fibroblasts include several mechanisms, as discussed recently by Chu [156]: inducing cell death through fibroblast-associated proteins, inhibiting binding to matrix protein, blocking signaling with endothelial cells, inhibiting proliferation and invasion, promoting apoptosis, inducing cellular senescence, and modulating fibroblast glucose metabolism. Similarly, inducing apoptosis in synovial macrophages and osteoclasts by using a chemotherapeutic-drug-loaded MMP9-cleavable, PEG- and RGD-peptide-modified PLGA nanoparticles were shown to be promising for treating severe inflammatory arthritis [157]. However, clinical studies are needed for further evaluation of these strategies.

Treatment with post-translationally modified peptides could attenuate inflammation. However, we do not know the actual autoantigens involved in arthritis, though several candidates have been proposed and tested so far. Similarly, antigen-specific tolerization by vaccination also has the same issue of identifying the causative autoantigens. Targeting specific cells like B cells with rituximab and inflammatory pathways like TNF-specific inhibitors improves joint inflammation. Still, it could pose various issues like treatment-associated infections, the economic burden to patients and society, treatment unresponsive patients, and so on. These treatment modalities are also not a cure for the disease.

Similarly, removing pathogenic antibodies from circulation or cleavage of Fc-carbohydrates can only decrease the inflammation at the effector phase of arthritis. However, using EVs from MSC could be a promising approach to reverse the inflammatory phenotypes in arthritis. Though there are many approaches for treating joint inflammation available both at clinical and pre-clinical stages, and more basic research needs to be conducted to understand the various disease pathways operating in the pre-RA phase before disease initiation and identifying the causative autoantigens and factors responsible for RA development.

## Data Availability

No new data were created or analyzed in this study. Data sharing is not applicable to this article.

## Abbreviations

Abca1	ATP binding cassette subfamily A member 1
ADCC	Antibody-dependent, cell-mediated cytotoxicity
ACPA	Anti-citrullinated protein antibodies
AMPK	Adenosine monophosphate-activated protein kinase
APC	Antigen-presenting cell
ATP	Adenosine triphosphate
Bcl-6	B cell lymphoma 6 transcription factor
BiP	Immunoglobulin-binding protein
CarP	Carbamylated protein
CCL	Chemokine Ligand
CCR2	C–C chemokine receptor type 2
CDC	Complement-dependent cytotoxicity
CIA	Collagen-induced arthritis
CTLA-4	Cytotoxic T lymphocyte antigen 4
CXCL	Chemokine (C–X–C motif) ligand
CII	Collagen type II
DC	Dendritic cells
DMARD	Disease-modifying, anti-rheumatic drug
E2F	Transcription factor
ECM	Extracellular matrix
EV	Extracellular vesicle
FcγR	Fc-gamma receptor
FLIP	FADD-like IL-1β-converting enzyme-inhibitory protein
FLS	Fibroblasts-like synoviocytes
FOXP3	Forkhead box P3
GATA3	GATA Binding Protein 3, a transcription factor
GC	Germinal center
G-CSF	Granulocyte colony-stimulating factor
GM-CSF	Granulocyte-macrophage colony-stimulating factor
GI	Gastro-intestinal
GPI	Glucose-6 phosphatase isomerase
HAPLN1	Hyaluronan and proteoglycan link protein 1
HLA	Human leukocyte antigen
IL	Interleukin
LAG-3	Lymphocyte-activation gene 3
K/BxN	Mice expressing T cell receptor transgene KRN and MHC class II Ag7
MAA	Malondialdehyde-acetaldehyde adduct
MHC	Major histocompatibility complex
MCP-1	Monocyte chemoattractant protein-1
MIP-1α	Macrophage inflammatory protein-1 alpha
miRNA	microRNA
MMP	Matrix metalloproteinase
MSC	Mesenchymal stem cell
mTORC1	Mammalian target of rapamycin complex 1
NADPH	Nicotinamide adenine dinucleotide phosphate
NETs	Neutrophil extracellular traps
NSAID	Nonsteroidal anti-inflammatory drug
OMV	Outer membrane vesicles
PAD	Peptidyl arginine deiminase
PDPs	platelet-derived microparticles
PEG	Polyethylene Glycol
PHD3	Prolyl hydroxylase 3
PLGA	Poly(lactic-co-glycolic acid)
PTM	Post-translational modification
PTPN22	Protein tyrosine phosphatase non-receptor type 22
RA	Rheumatoid arthritis
RANKL	Receptor activator of nuclear factor kappa beta (NFkB ligand)
RF	Rheumatoid factor
RGD	Arginylglycylaspartic acid
RORγt	RAR-related orphan receptor gamma—a transcription factor
ROS	Reactive oxygen species
SOCS3	Suppressor of cytokine signaling 3
Stat	Signal transducer and activator of transcription
T-bet	T-box family of transcription factor
TGF-β	Transforming growth factor-β
TNF-α	Tumor necrosis factor-alpha
Tfh cells	T follicular helper cells
ZAP-70	Zeta chain of T cell receptor-associated protein kinase 70
